# Microcystin-LR incorporated into colonic cells through probenecid-sensitive transporters leads to upregulated MCP-1 expression induced by JNK activation

**DOI:** 10.1016/j.toxrep.2022.04.019

**Published:** 2022-04-20

**Authors:** Yoshihito Koto, Hideaki Kawahara, Koichi Kurata, Keisuke Yoshikiyo, Ayumi Hashiguchi, Kunihiro Okano, Norio Sugiura, Kazuya Shimizu, Hidehisa Shimizu

**Affiliations:** a Graduate School of Natural Science and Technology, Shimane University, 1060 Nishikawatsu-Cho, Matsue, Shimane 690-8504, Japan; b Graduate School of Life and Environmental Science, Shimane University, 1060 Nishikawatsu-Cho, Matsue, Shimane 690-8504, Japan; c Institute of Agricultural and Life Sciences, Academic Assembly, Shimane University, 1060 Nishikawatsu-Cho, Matsue, Shimane 690-8504, Japan; d Institute of Environmental Systems Science, Academic Assembly, Shimane University, 1060 Nishikawatsu-Cho, Matsue, Shimane 690-8504, Japan; e Estuary Research Center, Shimane University, 1060 Nishikawatsu-Cho, Matsue, Shimane 690-8504, Japan; f Faculty of Bioresource Sciences, Akita Prefectural University, Akita 010-0195, Japan; g Faculty of Life and Environmental Sciences, University of Tsukuba, Ibaraki 305-8572, Japan; h Faculty of Life Sciences, Toyo University, Gunma 374-0193, Japan; i Raman Project Center for Medical and Biological Applications, Shimane University, 1060 Nishikawatsu-Cho, Matsue, Shimane 690-8504, Japan; j Project Center for Fortification of Local Specialty Food Functions, Shimane University, 1060 Nishikawatsu-Cho, Matsue, Shimane 690-8504, Japan; k Interdisciplinary Center for Science Research, Shimane University, 1060 Nishikawatsu-Cho, Matsue, Shimane 690-8504, Japan

**Keywords:** Cyanotoxin, Microcystin, MCP-1, JNK, Probenecid, Colon

## Abstract

Harmful algae that inhabit eutrophic lakes produce cyanotoxic microcystins. Therefore, the relationship between chronic exposure to microcystins via drinking water and organ disorders has been investigated. The present study aimed to determine whether representative microcystin-LR is involved in increased monocyte chemoattractant protein-1 (MCP-1) expression in rat colonic mucosa and enterocyte-like differentiated Caco-2 cells. The mRNA expression of MCP-1 was increased in the colons of rats administered with microcystin-LR, compared with controls. Furthermore, mRNA levels of MCP-1 expression significantly and positively correlated with those of Adhesion G Protein-Coupled Receptor E1 (ADGRE1; EMR1; F4/80), an indicator of macrophage infiltration, suggesting that increased MCP-1 expression induced by microcystin-LR promotes macrophage infiltration into the colon. Microcystin-LR increased MCP-1 expression in enterocyte-like differentiated Caco-2 cells, by activating c-Jun N-terminal kinase (JNK), but not extracellular signal-regulated kinase (ERK) or p38. The findings of transporter inhibitors indicated that microcystin-LR is incorporated into cells via ATP Binding Cassette (ABC) or solute carrier (SLC) transporters other than the organic anion transporting polypeptides (OATPs)1B1, 1B3, 2B1, and 1A2, which this leads to increased MCP-1 expression in the colon through activating JNK. Thus, increased MCP-1 expression induced by microcystin-LR might be a trigger for initiating tumorigenesis with inflammation in the colon because increased MCP-1 expression induces inflammation associated with macrophage infiltration into the colon, and chronic inflammation is associated with the initiation of tumorigenesis.

## Highlights

• Microcystin-LR upregulated colonic MCP-1 expression in rats.
• Increased MCP-1 expression correlated with accumulated macrophages in rat colon.
• Microcystin-LR evoked MCP-1 expression by activating JNK in cultured colon cells.
• Rifampicin was not involved in microcystin-LR-induced JNK activation.
• Probenecid suppressed JNK/MCP-1 pathway activation induced by microcystin-LR.

## Graphical Abstract

**Figure:**
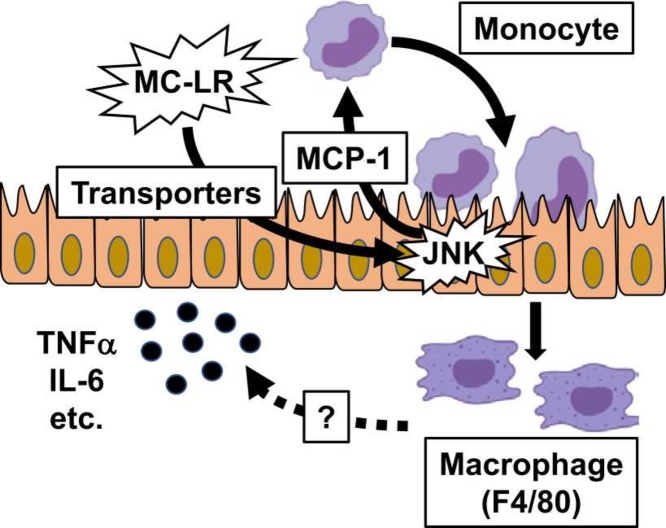

## 1 Introduction

**Fig. 1:**
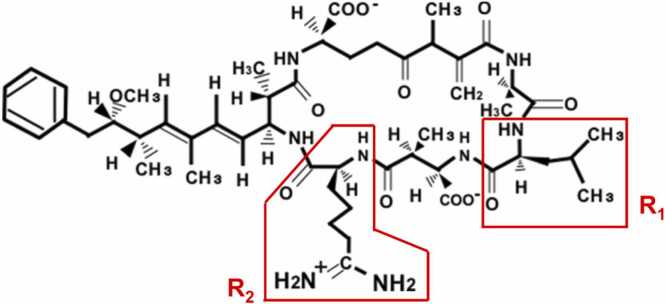
Chemical structure of microcystin-LR. Microcystin structure comprises five amino acids with minor variations (d-alanine, d-*erythro*-β-methyl aspartic acid, d-glutamic acid, *N*-methyldehydroalanine [Mdha], and 3-amino-9-methoxy-2,6,8-trimethyl-10-phenyldeca-4,6-dienoic acid [Adda]). They are characterized by one pair of variable l-amino acids at R_1_ and R_2_ locations in monocyclic heptapeptides. Microcystin-LR contains leucine (L) and arginine (R) residues at positions R_1_ and R_2_, respectively.

The eutrophication of surface waters leads to the development of harmful algal blooms (HAB) [1] that are associated with cyanobacterial species such as *Anabaena*, *Fischerella***,** *Gloeotrichia*, *Microcystis*, *Nodularia*, *Nostoc*, *Oscillatoria* and *Planktothrix* [2], [3], [4]. These cyanobacteria grow exponentially and produce secondary metabolites that are cyanotoxins [5]. Microcystins are ubiquitous cyanotoxins that have rapidly become a global health problem and are the only cyanobacterial toxins for which the World Health Organization (WHO) has established guideline values for drinking water [6]. Microcystins were detected in 28 of 30 subtropical lakes in eastern China during the summer of 2018, and the highest average microcystin occurring in Chaohu Lake was 26.7 μg/L [7]. In addition, water quality evaluations in the USA and Canada have detected microcystins in 80% of source and treated water samples [8], and microcystins comprising 60% of detectable cyanotoxins in brackish and fresh water in Europe [9]. Microcystins include 279 congeners with a common structure comprising five amino acids with minor variations (d-alanine, d-*erythro*-β-methyl aspartic acid, d-glutamic acid, *N*-methyldehydroalanine [Mdha], and 3-amino-9-methoxy-2,6,8-trimethyl-10-phenyldeca-4,6-dienoic acid [Adda]) and they are characterized by one pair of variable l-amino acids at the R_1_ and R_2_ locations in their monocyclic heptapeptides [10]. For instance, the most abundant and most toxic microcystin (microcystin-LR) contains leucine (L) and arginine (R) residues at the R_1_ and R_2_ locations, respectively [5] (Fig. 1), and it accounts for 46–99.8% of total microcystin concentrations in natural waters [11], [12]. Furthermore, the WHO has set an upper limit of 1 μg/L of microcystin-LR in drinking water and a tolerable daily intake (TDI) of 0.04 μg/kg [5].

Possible routes of microcystin exposure include the intake of contaminated drinking water, aquatic plants, fish, crops, and vegetables [13], and ingested microcystin-LR damages the liver, kidney, small intestine, nervous and reproductive systems [14]. However, the toxicological effects of long-term environmental exposure to microcystin-LR on the colon have not yet been investigated. Retrospective cohort studies in China have identified a significantly higher incidence of colorectal cancer (CRC) in populations that consume river or pond water compared with well or tap water, suggesting that microcystins are involved in colon carcinogenesis [15], [16]. In addition, CRC mortality rates in men increase with consistently increasing microcystin contents in water [16]. Microcystin concentrations are also positively associated with the incidence of CRC [16]. In addition to an association between microcystins and abnormal crypt foci (ACF) in the colons of male C57BL/6 J mice induced by azoxymethane (AOM) [17], such findings in humans indicated an association between the chronic ingestion of water contaminated with microcystins and an increased incidence of CRC, but the involved mechanisms have remained unclear.

Microcystins that enter cells specifically interact with and inhibit the serine/threonine protein phosphatases 1 and 2A (PP1 and PP2A), via a two-step mechanism. The first is the formation of a non-covalent bond between the Adda side chain and the glutamyl carboxyl, followed by that of an irreversible covalent bond between the Mdha group of the toxin and cysteine residue(s) in protein phosphatase. Microcystins are thought to lead to organ disorders via hyperphosphorylated PP1 and PP2A target molecules [18], [19]. However, microcystins cannot readily enter target cells; thus, they require an uptake mechanism. The organic anion transporting polypeptides (OATP)1B1, 1B3, 2B1, and 1A2 have been extensively studied with respect to the cellular uptake of microcystins. Since the expression of OATP1B1 and OATP1B3 is considered specific to the liver [20], their contribution to the intestinal uptake of microcystins may be negligible. Furthermore, OATP2B1 is not involved in microcystin uptake although it is apparently expressed in the intestines [21]. The expression of OATP1A2 in the intestine and its function in the cellular uptake of microcystins have not yet been clarified [21].

Potent monocyte chemotactic protein-1 (MCP-1; also known as C-C motif chemokine ligand 2; CCL2) belongs to a subfamily of C-C chemokines [22]. Its increased expression contributes to the development of CRC by recruiting and infiltrating monocytes into the colorectum, where they differentiate into macrophages [23]. The expression of *MCP-1* mRNA correlates with the abundance of large intestinal polyps in Apc^*Min*/+^ mice that are the most predominant models for studying cancers involving the gastrointestinal tract [24], and MCP-1 is also implicated in the development and progression of CRC in other mouse strains [23], [25]. Furthermore, the expression of MCP-1 is associated with the abundance of macrophages in the colorectum and CRC tumorigenesis in humans [26]. The expression of MCP-1 induced by microcystin-LR is upregulated in models of inflammatory bowel disease that induces CRC development [27], [28]. However, a link between chronic microcystin intake by healthy animals and increased colonic MCP-1 expression has not yet been established. Furthermore, the transporters involved in microcystin uptake and the mechanism through which microcystin-LR upregulates colonic MCP-1 expression await clarification. Thus, the present study aimed to determine whether *MCP-1* mRNA expression increases in the colons of healthy rats exposed to long-term environmental levels (10 μg/L) of microcystin-LR via drinking water provided *ad libitum* for 7 weeks, and to clarify the mechanism through which microcystin-LR causes increased MCP-1 expression in the differentiated Caco-2 cell models of intestinal epithelial cells.

## 2 Materials and methods

### 2.1 Materials and reagents

Antibodies and other reagents were obtained from the following suppliers: anti-MCP-1 (Abcam, Cambridge, UK); anti-β-actin (C4) (Santa Cruz Biotechnology, Inc., Dallas, TX, USA); anti-phospho-extracellular signal-regulated kinase (ERK) (Thr202/Tyr204), anti-phospho-p38 (Thr180/Tyr182), anti-phospho-c-Jun N-terminal kinase (JNK) (Thr183/Tyr185), anti-rabbit and anti-mouse IgG-HRP-linked antibodies (Cell Signaling Technology, Inc., Danvers, MA, USA); rifampicin, Protease Inhibitor Cocktail (EDTA free) (100x), and Phosphatase Inhibitor Cocktail (all from Nacalai Tesque Inc., Kyoto, Japan); Microcystin-LR, penicillin-streptomycin solution (x100), Dulbecco’s modified Eagle's medium (DMEM) supplemented with low glucose, and SP600125 (all from Wako Pure Chemical Industries Ltd., Osaka, Japan); probenecid (Sigma-Aldrich, St Louis, MO, USA), and fetal bovine serum (FBS; Biowest S.A.S., Nuaillé, France).

### 2.2 Animal experiments

The Animal Care and Use Committee of Shimane University approved all animal experiments and procedures (Approval no: MA28–1 and MA31–3). The housing, husbandry and handling of rats complied with the Institutional Regulations of Shimane University that were established in accordance with the Act on Welfare and Management of Animals (Act No. 105) and relevant standards and guidelines in Japan. Five-week-old male WKAH/HkmSlc rats (Japan SLC, Inc., Hamamatsu, Japan) were housed in individual plastic cages in an air-conditioned room at 22 °C ± 2 °C with 55% ± 5% humidity under an automated light cycle (lights on at 08:00 and off at 20:00) throughout the study. The rats were given the AIN-93 G diet without *t*-butylhydroquinone (Table 1) and deionized water *ad libitum*. After acclimation for one week, the rats were assigned to control or experimental groups (n = 11 each) that were given free access to deionized water (control) or microcystin-LR (10 μg/L) in deionized water for 7 weeks. Thereafter, the rats were euthanized by exsanguination under anesthesia with 5% isoflurane for induction and 2% for maintenance via a nose cone, then colonic mucosa was scraped onto glass slides and stored at − 80 °C.

**Table 1:** Dietary ingredients.

	Ingredients g/kg diet
Cornstarch^a^	397.5
Casein^b^	200.0
Dextrin^c^	132.0
Sucrose^d^	100.0
Soybean oil^e^	70.0
Mineral mixture^f^	35.0
Vitamin mixture^g^	10.0
Choline bitartrate^h^	2.5
L-Cystine^h^	3.0
Cellulose^i^	50.0

a Amylalpha CL (Chuo-Shokugyou Co. Ltd., Inazawa, Japan).

b Milk casein (CLEA Japan, Inc., Tokyo, Japan).

c TK-16 (Matsutani Chemical Industry Co., Ltd., Hyogo, Japan).

d Nippon Beet Sugar Mfg. Co. Ltd., Tokyo, Japan.

e Oriental Yeast CO., Ltd., Tokyo, Japan.

f AIN-93 mineral mixture (MP Biomedicals LLC, Santa Ana, CA, USA).

g AIN-93 vitamin mixture (CLEA Japan, Inc., Tokyo, Japan).

h Wako Pure Chemical Industries Ltd., Tokyo, Japan).

i Ceolus (Asahi Kasei Chemicals Co. Ltd., Tokyo, Japan).

### 2.3 Cell culture

The human colon cancer cell line Caco-2 (RIKEN Cell Bank, Tsukuba, Japan) spontaneously differentiates into an enterocyte-like phenotype and serves as a model for intestinal epithelial cells [29]. We cultured Caco-2 cells in low-glucose DMEM supplemented with 10% FBS, 100 U/mL penicillin and 100 μg/mL streptomycin at 37 °C under a 7% CO_2_ atmosphere for 28 days as we previously described [30]. The medium was changed every 2 days, and the cells were incubated in serum-free DMEM for 24 h before all experiments. Dimethyl sulfoxide (DMSO) was the control vehicle, and the solvent (final concentration 0.1% v/v) for microcystin-LR, SP600125, rifampicin, and probenecid.

### 2.4 Quantitative real-time PCR

Serum-starved enterocyte-like differentiated Caco-2 cells were incubated with or without SP600125 (5 μM), rifampicin (10 μM), and probenecid (500 μM) for 30 min, then stimulated with or without microcystin-LR (10 nM) for various periods. Total RNA was extracted from the cells and colonic mucosa was scraped using Sepasol-RNA I Super G (Nakalai Tesque Inc., Kyoto, Japan) and RNeasy Mini Kits (QIAGEN, Hilden, Germany), respectively. First-strand cDNA was synthesized from template RNA (1 μg) using PrimeScript™ RT Master Mix (Perfect Real Time) (Takara Bio Co. Inc., Shiga, Japan). Quantitative real-time PCR proceeded using TB Green™ Premix Ex Taq™ II (Tli RNaseH Plus), a Thermal Cycler Dice Real Time System III (all from Takara Bio Co. Inc.) as described by the manufacturer, and oligonucleotide primers (Table 2). Amplicons were quantified using a calibration curve of serial dilutions of known DNA concentrations and quantitation cycle (Cq) values plotted against log sample concentrations, and mRNA expression was measured as ratios of rat or human ribosomal protein lateral stalk subunit P0 (*RPLP0*) mRNA as an internal standard.

**Table 2:** Forward and reverse primer sequences of target genes.

Target genes	Primers (5′ →3′)
rat *MCP-1*	F: TTAGAAAACTGGACCAGAACCAA
	R: GCATTAGCTTCAGATTTATGGGT
rat *TNF*	F: CCCTCACACTCAGATCATCTTCT
	R: GCTACGACGTGGGCTACGG
rat *IL6*	F: TAGTCCTTCCTACCCCAACTTCC
	R: TTGGTCCTTAGCCACTCCTTC
rat *ADGRE1*	F: TCAGGGCCCAGGAGTGGAA
	R: GTGCAGACTGAGTTAGAACCACA
rat *RPLP0*	F: GCTCCAAGCAGATGCAGCA
	R: CCGGATGTGAGGCAGCAG
human *MCP-1*	F: CAAGCAGAAGTGGGTTCA
	R: GGGAAAGCTAGGGGAAAATAAG
human *RPLP0*	F: CGACCTGGAAGTCCAACTAC
	R: ATCTGCTGCATCTGCTTG

### 2.5 Immunoblotting

Serum-starved enterocyte-like differentiated Caco-2 cells were incubated with or without SP600125 (5 μM), rifampicin (10 μM), and probenecid (500 μM) for 30 min, then stimulated with or without microcystin-LR (10 nM) for various periods. The cells were lysed in 1% NP-40 buffer containing 150 mM NaCl, 50 mM Tris-HCl, 10% glycerol, Protease Inhibitor Cocktail, and Phosphatase Inhibitor Cocktail), resolved by SDS-PAGE on polyacrylamide gels, then blotted onto Immobilon-P polyvinylidene fluoride membranes (Millipore Inc., Bedford, MA, USA). We detected MCP-1, phospho-ERK, phospho-p38, phospho-SAPK/JNK, and β-actin using anti-MCP-1 (1:1000-diluted), anti-phospho-ERK (1:5000-diluted), anti-phospho-p38 (1:1000-diluted), anti-phospho-SAPK/JNK (1:1000-diluted) and anti-β-actin (1:5000-diluted) antibodies, respectively. The intensity of blotted proteins was visualized using enhanced Chemi-Lumi One L (Nakalai Tesque Inc.) and analyzed using an ImageQuant™ LAS 4000 densitometer and its integrated ImageQuant™ software (GE Healthcare Life Sciences Corp., Uppsala, Sweden). Levels of MCP-1 and phospho-JNK bands normalized to the amount of β-actin are expressed as ratios (fold increase) of control value.

### 2.6 Statistical analysis

**Fig. 2:**
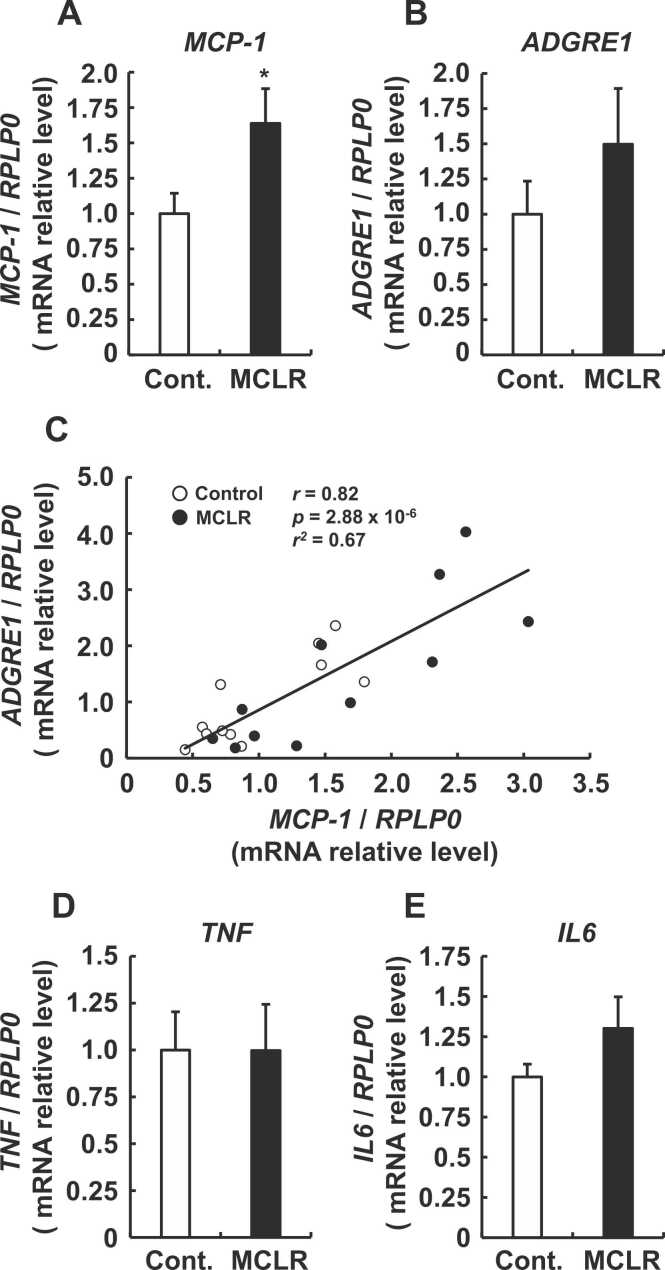
Expression of inflammatory-related genes, and *ADGRE1* mRNA and correlations between mRNA levels of *MCP-1* and *ADGRE1* in colonic mucosa. Expression of *MCP-1* (A) and *ADGRE1* (B) mRNA in colonic mucosa measured by real-time PCR. Data are shown as means ± SE (controls and microcystin-LR groups, n = 11 each; *P < 0.05 vs. control). (C) Relationships between *MCP-1* and *ADGRE1* mRNA expression determined using Pearson correlation tests (control and microcystin-LR groups, n = 11 each). Expression of *TNF* (D) and *IL6* (E) mRNA in colonic mucosa measured by real-time PCR. Data are shown as means ± SE (controls and microcystin-LR groups, n = 11 each; *P < 0.05 vs. control). Messenger RNA expression was calculated as ratios of rat *RPLP0* mRNA. Cont.: control; MCLR: microcystin-LR.

**Fig. 3:**
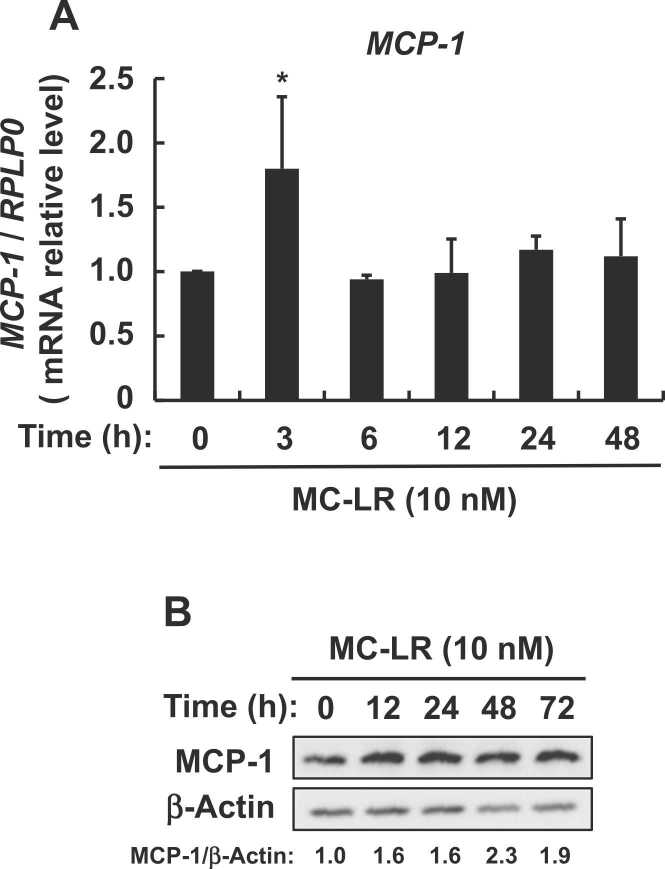
Effects of microcystin-LR on MCP-1 expression in enterocyte-like differentiated Caco-2 cells. Serum-starved enterocyte-like differentiated Caco-2 cells were incubated with or without microcystin-LR (10 nM) for indicated periods, then (A) *MCP-1* mRNA expression was measured by real-time PCR and (B) cell lysates were immunoblotted using anti-MCP-1 and anti-β-actin antibodies. Data (A) are expressed as means ± SE of three independent experiments. *P < 0.05 vs. DMSO vehicle (Control). Messenger RNA expression was calculated as ratios of human *RPLP0* mRNA. MC-LR: microcystin-LR.

**Fig. 4:**
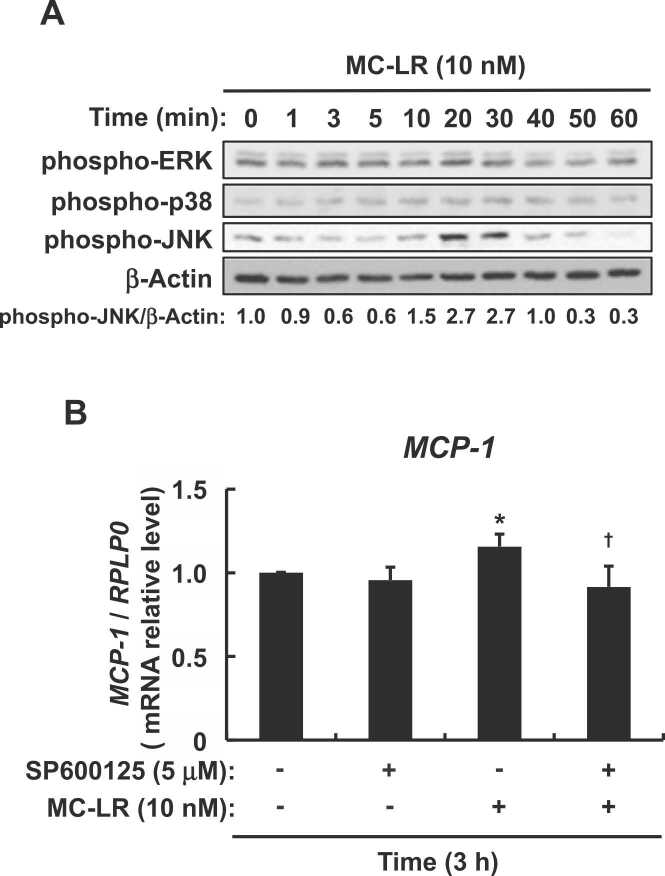
Effects of microcystin-LR on relationship between MAPK and MCP-1 expression in enterocyte-like differentiated Caco-2 cells. (A) Serum-starved enterocyte-like differentiated Caco-2 cells were incubated with or without microcystin-LR (10 nM) for indicated periods, then cell lysates were immunoblotted against anti-phospho-ERK, anti-phospho-38, anti-phospho-SAPK/JNK, and anti-β-actin antibodies. (B) Serum-starved enterocyte-like differentiated Caco-2 cells were incubated with or without JNK inhibitor SP600125 (5 μM) for 30 min, followed by microcystin-LR (10 nM) for 3 h, then *MCP-1* mRNA expression was measured by real-time PCR. Data are expressed as means ± SE of five independent experiments for (B). *P < 0.05 vs. DMSO vehicle (Control). Messenger RNA expression was calculated as ratios of human *RPLP0* mRNA. MC-LR: microcystin-LR.

**Fig. 5:**
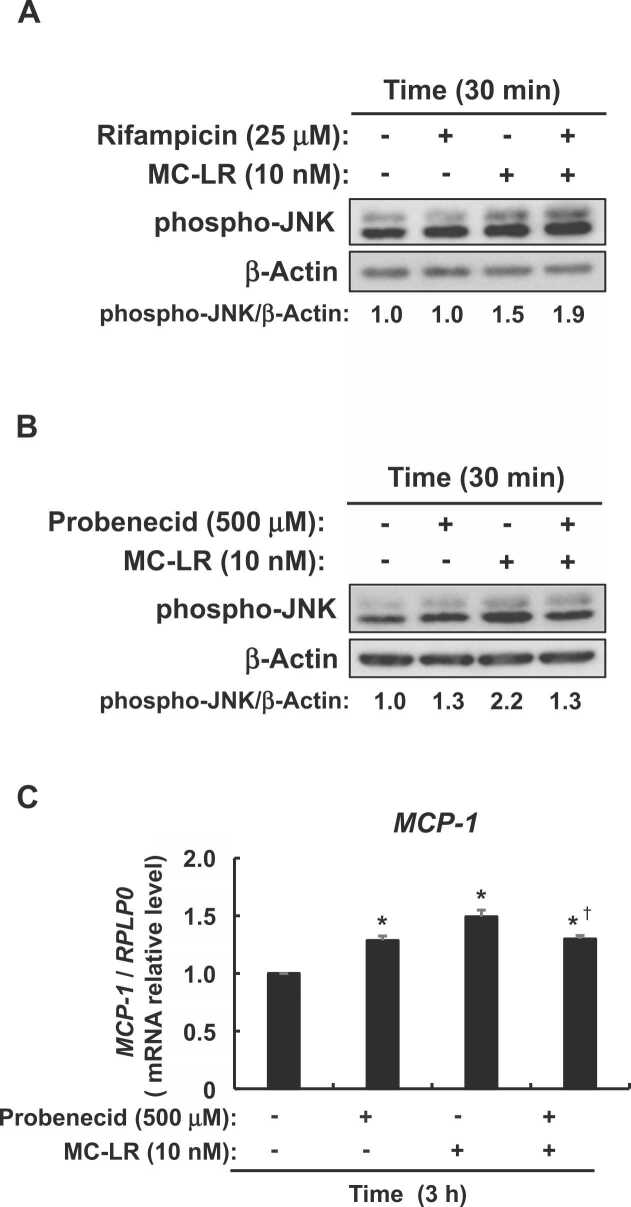
Effects of transporter inhibitors on microcystin-LR-induced JNK activation and MCP-1 expression in enterocyte-like differentiated Caco-2 cells. (A) Serum-starved enterocyte-like differentiated Caco-2 cells were incubated with or without rifampicin (10 μM) for 30 min followed by microcystin-LR (10 nM) for 30 min, then cell lysates were immunoblotted against anti-phospho-SAPK/JNK and anti-β-actin antibodies. (B) Serum-starved enterocyte-like differentiated Caco-2 cells were incubated with or without probenecid (500 μM) for 30 min, followed by microcystin-LR (10 nM) for 30 min, then cell lysates were immunoblotted using anti-phospho-SAPK/JNK and anti-β-actin antibodies. (C) Serum-starved enterocyte-like differentiated Caco-2 cells were incubated with or without probenecid (500 μM) for 30 min, followed by microcystin-LR (10 nM) for 3 h, then *MCP-1* mRNA expression was measured by real-time PCR. Data are expressed as means ± SE of four independent experiments for (C). *P < 0.05 vs. DMSO vehicle (Control). Messenger RNA expression was calculated as ratios of human *RPLP0* mRNA. MC-LR: microcystin-LR.

Results are expressed as means ± SE. Data were statistically analyzed by Student *t*-tests (Fig. 2A, B, D, and E), Pearson correlations (Fig. 2C), Dunnett (Fig. 3A), and Tukey-Kramer (Fig. 4B and Fig. 5C) tests using Excel 2011 (Microsoft Corp., Redmond, WA, USA) and Statcel 4 (OMS Publishing Co., Saitama, Japan). Values were considered statistically significant at *p* < 0.05.

## 3 Results

### 3.1 Expression of MCP-1 mRNA is upregulated in colons of rats administered with microcystin-LR

Among plants that treat water from Lake Erie and other locations in Ohio and neighboring states for consumption, the maximum microcystin concentration detected in finished drinking water was ~10 μg/L at the Celina plant [31]. Based on that finding, the rats were non-invasively exposed to an environmentally appropriate concentration of microcystin-LR (10 μg/L) in drinking water provided *ad libitum* for 7 weeks as described [32]. None of the rats died; and initial and final body weight as well as total food intake at 7 weeks did not significantly differ between the exposed and control groups (Table 3). The mRNA expression of *MCP-1* was increased in the colonic mucosa of rats exposed to microcystin-LR compared with the controls (Fig. 2A). This is considered to be the most important chemokine in terms of recruiting macrophages and levels of Adhesion G Protein-Coupled Receptor E1 (ADGRE1; EMR1; F4/80), an indicator of macrophage infiltration, are reduced in polyps of Apc^*Min*/+^/MCP-1^−/−^ mice [24]. Therefore, we examined whether macrophages also infiltrate the colonic mucosa of healthy rats using *ADGRE1* mRNA expression as an indicator. Fig. 2B shows that the average mRNA expression of *ADGRE1* tended to be high in the rats given microcystin-LR but did not significantly differ from that in the colonic mucosa of control rats that consumed water. However, because the mean values of *MCP-1* and *ADGRE1* were elevated in rats exposed to microcystin-LR, we predicted that *MCP-1* expression would correlate with macrophage infiltration in the colonic mucosa. Fig. 2C shows that *MCP-1* and *ADGRE1* mRNA expression significantly and positively correlated (*r* = 0.82, *p* = 2.88 x 10^-6^, *r*^*2*^ = 0.67). That is, *MCP-1* expression was reflected in the amount of macrophage infiltration in the rat colonic mucosa. Therefore, microcystin-LR may help to promote macrophage infiltration by increasing *MCP-1* expression in the colonic mucosa. Furthermore, since *TNF* (tumor necrosis factor α: TNFα) and *IL6* (interleukin-6: IL-6) are established inflammatory marker genes along with *MCP-1*, we compared their expression between the two groups of rats. Fig. 2D and E show that the expression of *TNF* and *IL6* mRNA did not significantly change in either group (Fig. 1A). Therefore, the increased expression of *MCP-1* may lead to macrophage accumulation via elevated *MCP-1* expression, although increased *TNF* and *IL6* expression did not significantly change in the colons of rats administered with 10 μg/L microcystin-LR for 7 weeks.

**Table 3:** Body weight gain and food intake.

	Control	Microcystin-LR	*p*
Initial BW (g)	187.8 ± 7.18	186.4 ± 6.95	NS
Final BW (g)	378.8 ± 12.42	388.4 ± 10.35	NS
Total food intake (g)	884.1 ± 25.82	888.0 ± 20.20	NS

BW, body weight.

### 3.2 Microcystin-LR leads to upregulated MCP-1 expression in enterocyte-like differentiated Caco-2 cells

We examined whether 10 nM microcystin-LR, which corresponds to 10 μg/L in rats based on its molecular weight of 995.17, would induce the expression of *MCP-1* mRNA in enterocyte-like differentiated Caco-2 cells as models of intestinal epithelial cells. Fig. 3A and B shows that microcystin-LR increased *MCP-1* mRNA and MCP-1 protein expression. Therefore, increased MCP-1 expression induced by microcystin-LR was observed in rat colonic mucosa as well as in enterocyte-like differentiated Caco-2 cells.

### 3.3 Activation of JNK induced by microcystin-LR leads to upregulated MCP-1 expression in enterocyte-like differentiated Caco-2 cells

The activated mitogen-activated protein kinases (MAPKs) ERK and JNK are involved in increased MCP-1 expression in renal proximal tubular cells [33]. Therefore, we predicted that at least one of ERK, p38, and JNK would also be involved in the regulation of MCP-1 expression in enterocyte-like differentiated Caco-2 cells. Fig. 4A shows that microcystin-LR significantly activated JNK and very slightly activated ERK and p38. We therefore assessed the relationship between the significantly activated JNK and increased MCP-1 expression. We used *MCP-1* mRNA expression as an indicator because it was reflected as increased protein levels when induced by microcystin-LR (Fig. 3A and B). Fig. 4B shows that SP600125, a JNK inhibitor, suppressed the *MCP-1* mRNA expression induced by microcystin-LR. These results showed that JNK activation induced by microcystin-LR is involved in the upregulation of MCP-1 expression in enterocyte-like differentiated Caco-2 cells.

### 3.4 Probenecid-sensitive transporter participates in increased MCP-1 expression by activating JNK induced by microcystin-LR in enterocyte-like differentiated Caco-2 cells

We investigated relationships between JNK activation induced by microcystin-LR and transporters in enterocyte-like differentiated Caco-2 cells. Fig. 5A shows that rifampicin, an OATP1B1, OATP1B3, OATP2B1, and OATP1A2 inhibitor, did not repress microcystin-LR-induced JNK activation. In contrast, probenecid, an inhibitor of the ATP Binding Cassette (ABC) and solute carrier (SLC) transporters, suppressed JNK activation induced by microcystin-LR (Fig. 5B) and *MCP-1* expression (Fig. 5C). Thus, probenecid-sensitive ABC and SLC transporters other than OATP1B1, 1B3, 2B1, and 1A2 are involved in the JNK/MCP-1 pathway activation induced by microcystin-LR.

## 4 Discussion

**Fig. 6:**
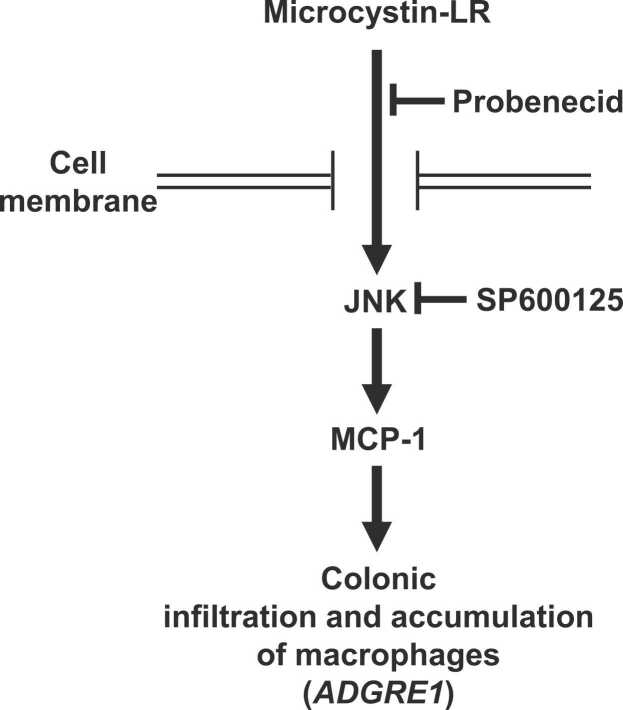
Schematic representation of microcystin-LR functions in colon. Activation of JNK induced by microcystin-LR through probenecid-sensitive transporters accompanied by increased colonic MCP-1 expression, subsequently causes macrophage infiltration and accumulation in colon.

The graphical abstract and Fig. 6 summarize the present findings. We suggest that increased expression of colonic MCP-1 is driven by JNK activation induced by microcystin-LR taken into cells by probenecid-sensitive transporters other than OATP1B1, 1B3, 2B1, and 1A2, resulting in colonic macrophage accumulation. Therefore, because increased MCP-1 expression might be the first step in the microcystin-LR-induced tumorigenesis of CRC, the present findings provide important clues about the mechanism of its development and progression.

We could not confirm that microcystin-LR increased *TNF* and *IL6* expression in the colonic mucosa of rats. However, the expression of *ADGRE1*, an indicator of macrophage infiltration, and *MCP-1* significantly correlated in the colonic mucosa. Therefore, longer exposure may lead to further macrophage infiltration and eventually induce intestinal inflammation with increased expression of macrophage-derived TNFα and IL-6 in the rat colonic mucosa. The increased expression of TNFα, a representative pro-inflammatory cytokine in CRC tissues, participates in the induction of CRC proliferation [34] and in the epithelial-mesenchymal transition, which plays an essential role in accelerating CRC invasion and metastasis [35]. Therefore, inhibiting the elevated colonic MCP-1 expression induced by microcystin-LR may prevent the accumulation of macrophages infiltrating the colon via MCP-1 and consequently suppress TNFα production derived from macrophages that have already accumulated in the colon. This may prevent the development of CRC and attenuate its progression. Furthermore, since increased colonic inflammation is a major factor in the development and progression of hepatocellular carcinoma (HCC) [36] and microcystin-LR is considered to be a carcinogenic promoter of HCC [37], we speculate that the increased expression of colonic MCP-1 determined herein is involved in the effects of microcystins on HCC as well as CRC.

Since probenecid, but not rifampicin, suppressed JNK activation and increased *MCP-1* expression induced by microcystin-LR, we propose that probenecid-sensitive ABC and SLC transporters other than 1B1, 1B3, 2B1, and 1A2 are involved in the cellular uptake of microcystin-LR. In addition to these four transporters, others that are expressed in both human colon and Caco-2 cells are monocarboxylate transporter 1 (MCT1), peptide transporter 1 (PEPT1), cation/carnitine transporter 2 (OCTN2), and organic cation transporters 1 and 3 (OCT1 and OCT3), OATP3A1, and 4A1 [20], [38], [39], [40]. However, since MCT1 is a lactate transporter, the ketone bodies acetoacetate and β-hydroxybutyrate, the branched-chain keto-acids such as α-ketoisocaproate formed from transamination of amino acids, and the short-chain fatty acids such as acetate and butyrate [41] might not be involved in the cellular uptake of microcystin-LR. Moreover, because PEPT1 is a transporter for the uptake of dipeptides and tripeptides [42], the monocyclic heptapeptide microcystin-LR [10] might not be a substrate for this transporter. In addition, since OATP1B1 and 1B3 that are involved in the cellular uptake of microcystins are organic anion transporters, the organic cation transporters, OCT1, OCT3, and OCTN2, are probably also not involved in cellular microcystin-LR uptake [43]. Therefore, OATP3A1 and/or OATP4A1, which are the same organic cation transporters as OATP1B1 and 1B3, are the most likely microcystin-LR transporters in the colon. This notion is similar to that of Zeller et al. [39]. However, further studies should determine whether OATP3A1 and/or 4A1 are involved in the cellular uptake of microcystin-LR.

The phosphorylation of JNK, a stress-inducible MAP kinase, was induced not only by microcystin-LR but also by probenecid in enterocyte-like differentiated Caco-2 cells. Probenecid also slightly activates another stress-inducible MAP kinase p38 in the normal lung epithelial fibroblast cell line RWI38 [44]. This might be because inactivation of the probenecid-sensitive transporter blocked the entry of compounds necessary for homeostasis into the cells, which caused intracellular stress. In addition, probenecid suppressed comparable amounts of microcystin-LR-dependent and independent JNK phosphorylation. These levels of JNK phosphorylation were also reflected in *MCP-1* expression. Therefore, we consider that JNK phosphorylation and increased *MCP-1* expression induced by microcystin-LR were suppressed by inactivating the probenecid-sensitive transporter. Overall, the present findings indicated that microcystin-LR is uptaken by cells via probenecid-sensitive transporters to activate JNK with subsequent increased *MCP-1* expression in enterocyte-like differentiated Caco-2 cells.

Since microcystin-LR inhibited PP1A and PP2A, we considered that ERK, p38, and JNK may be activated in enterocyte-like differentiated Caco-2 cells. However, only JNK was significantly activated. A similar situation has been identified the c-Src/p130Cas-mediated signaling pathway in response to H_2_O_2_ in vascular smooth muscle cells [45]. Furthermore, H_2_O_2_ suppresses the activity of PP2A but not that of PP1 [46] and c-Src is directly associated with and negatively regulated by PP2A [47]. That is, PP2A activity repressed by H_2_O_2_ leads to c-Src activation. Based on these findings, the JNK activation determined herein might result from activation of the c-Src/p130Cas-mediated signaling pathway by microcystin-LR acting on PP2A but not PP1.

Since microcystins are thought to act on PP1 and PP2A for extended periods and inhibit their activity, target molecules of these two phosphatases are likely to remain phosphorylated by microcystins for extended periods. However, we found that the phosphorylation of JNK was transient. Negative regulation of intracellular signaling involves not only serine/threonine phosphatases such as PP1 and PP2A but also protein tyrosine phosphatases (PTPs). For example, the c-Src/p130Cas pathway described above is negatively regulated by PTP-PEST, a scaffold PTP, resulting in the attenuated activation of downstream signaling transduction molecules [48], [49]. Therefore, considering negative regulation not only by serine/threonine phosphatases but also by PTPs, the transient phosphorylation of JNK induced by microcystin-LR was hardly unusual.

## 5 Conclusion

The activation of JNK induced by microcystin-LR through probenecid-sensitive transporters other than OATP1B1, 1B3, 2B1, and 1A2 is accompanied by increased MCP-1 protein expression in the colon. Understanding the mechanism of microcystin uptake into the colon is necessary to clarify the molecular mechanisms of its activities. Therefore, if probenecid-sensitive transporters involved in the uptake of microcystins into the colon are identified and dietary components or effective pharmacological approaches that inhibit the activation of such transporters can be found, the development of CRC and other diseases such as HCC caused by microcystins might be preventable.

## CRediT authorship contribution statement

**Yoshihito Koto:** Investigation, Writing – review & editing, Project administration. **Hideaki Kawahara:** Investigation. **Koichi Kurata:** Investigation. **Keisuke Yoshikiyo:** Investigation. **Ayumi Hashiguchi:** Writing – review & editing. **Kunihiro Okano:** Conceptualization. **Norio Sugiura:** Conceptualization. **Kazuya Shimizu:** Conceptualization, Writing – review & editing. **Hidehisa Shimizu:** Conceptualization, Investigation, Writing –- original draft, Writing – review & editing, Supervision, Funding acquisition.

## Declaration of Competing Interest

No potential conflicts of interest are reported by the authors.
